# Out-of-hospital cardiac arrest and survival in a patient with Noonan syndrome and multiple lentigines: a case report

**DOI:** 10.1186/s13256-019-2096-6

**Published:** 2019-06-15

**Authors:** Christian Eichhorn, Inga Voges, Piers E. F. Daubeney

**Affiliations:** 1 Department of Paediatric Cardiology, Royal Brompton and Harefield NHS Foundation Trust, London, UK; 2 Department of Congenital Heart Disease and Paediatric Cardiology, University Hospital Schleswig-Holstein, Campus Kiel, Arnold-Heller-Str. 3, 24105 Kiel, Germany; 3 National Heart and Lung Institute, Imperial College, London, UK

**Keywords:** Cardiac arrest, LEOPARD syndrome, Noonan syndrome with multiple lentigines, Cardiopulmonary resuscitation

## Abstract

**Background:**

A 9-year-old Arabic boy attending middle school presented with an out-of-hospital cardiac arrest due to ventricular fibrillation recorded by Holter electrocardiographic monitoring. He had a background history of Noonan syndrome with multiple lentigines (also known as LEOPARD syndrome), a rare condition of autosomal dominant inheritance with approximately 200 cases reported worldwide.

**Case presentation:**

Apart from characteristic features, the boy was known to have asymmetric septal hypertrophy with a maximum wall thickness of 24 mm measured by cardiovascular magnetic resonance imaging. A day prior to the event, he attended cardiology follow-up at our institution, and Holter monitoring was commenced. Following cardiopulmonary resuscitation by bystanders and paramedics, he reverted back into sinus rhythm after a total downtime of 24 min. He was initially treated in the intensive care unit and underwent implantable cardioverter defibrillator implantation. He has made a full recovery and remains at the top of his class.

**Conclusion:**

This case demonstrates that sudden cardiac arrest in patients with secondary forms of hypertrophic cardiomyopathy is not necessarily protected by apparently favorable phenotypes and that events may be preceded by non-sustained ventricular tachycardia observed by Holter monitoring. Implantable cardioverter defibrillator implantation plays a critical role in both primary and secondary prevention in patients at high risk of out-of-hospital cardiac arrest.

## Background

To our knowledge, this is the first reported case of a 9-year-old patient with Noonan syndrome with multiple lentigines (NSML) and out-of-hospital cardiac arrest (OHCA) during ambulatory electrocardiographic monitoring. Cardiac involvement, in particular hypertrophic cardiomyopathy (HCM), is detected in the vast majority of patients with NSML. This case is a characteristic example of the malignancy of HCM in some young patients and the need for early implantable cardioverter defibrillator (ICD) implantation. The question whether concomitant syndromes confer an additional risk is yet to be fully investigated. In a previous study by Limongelli *et al.* [1], 4 (21%) of 19 patients with NSML with left ventricular (LV) hypertrophy experienced sudden cardiac arrest or died during follow-up. Limongelli *et al*. also reported the sudden cardiac death (SCD) of a 9-year-old boy with NSML and severe non-obstructive HCM [2]. This patient had a non-sustained ventricular tachycardia (VT) seen on a Holter electrocardiogram (ECG) prior to the event [2]. Another case report, by Woywodt *et al.*, demonstrated that sudden cardiac arrest in patients with NSML can happen in less affected patients with NSML [3].

Our patient’s case is unique not only because the progression of sinus rhythm to ventricular fibrillation (VF) was captured during ambulatory ECG monitoring but also because the patient made a full recovery despite a poor prognosis. This case report also highlights the principles used in the care of this patient and raises the importance of educational campaigns for trained and untrained staff in the use of bystander cardiopulmonary resuscitation (CPR). Additionally, it demonstrates the importance of managing the complications of complex congenital cardiac disease in specialized centers.

## Case presentation

**Fig. 1:**
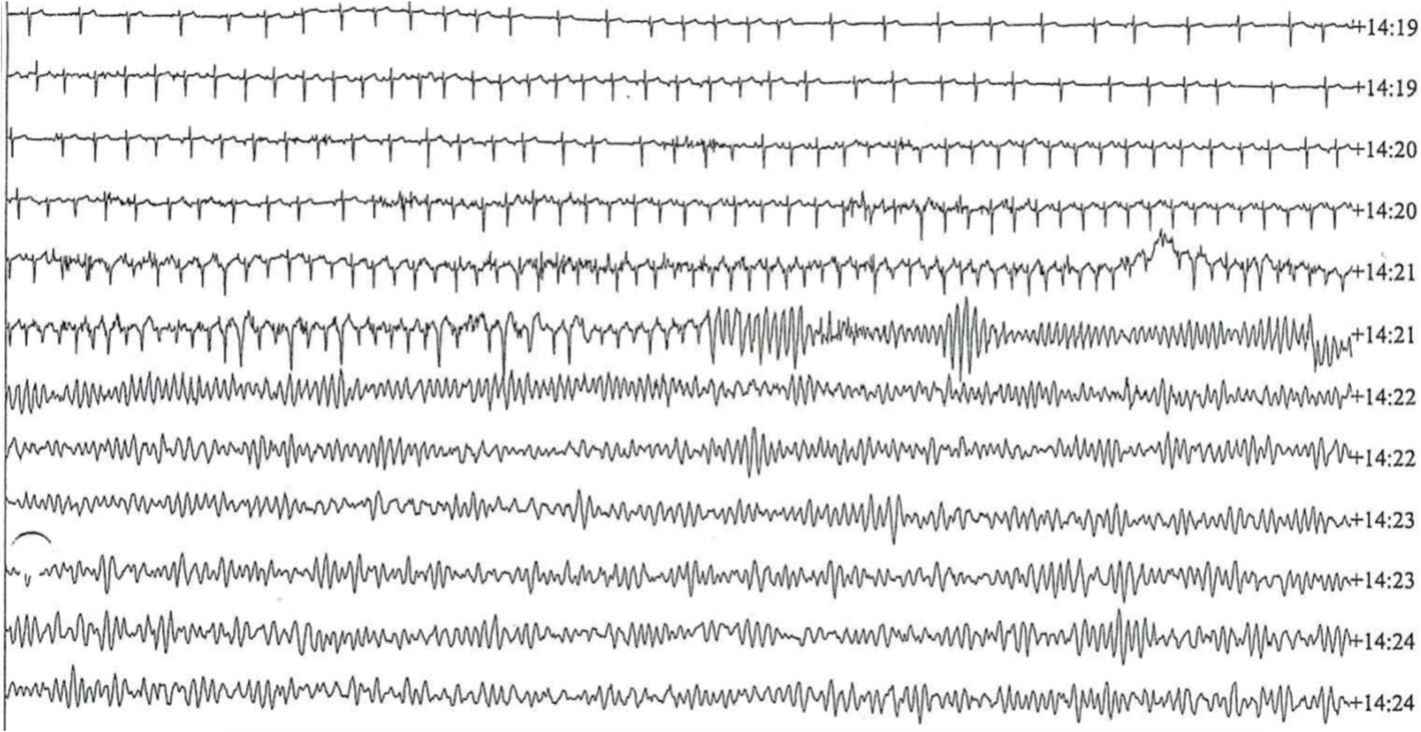
Holter monitor recording during the time of the event showing an increasing number of ectopic beats and progression from sinus rhythm to torsades de pointes and ventricular fibrillation

A 9-year-old Arabic boy attending middle school experienced OHCA, witnessed by his fellow students, during a physical education lesson. Coincidentally, he had been fitted with a Holter monitor at the time of the event (Fig. 1). His medical history comprised a fetal diagnosis of NSML (formerly known as LEOPARD syndrome due to *PTPN11* gene mutation) with characteristic features of hypertelorism, low-set ears with prominent pinna bilaterally, downward-slanting palpebral fissures, slight visual disturbances, multiple freckles and lentigines on his face and body, and mild pulmonary stenosis and asymmetric septal hypertrophy diagnosed post-delivery. He was started on regular doses of β-blockers after birth and was receiving bisoprolol 2.5 mg once daily at the time of the event. Cardiac magnetic resonance (CMR) imaging 4 months prior to the event showed a maximum septal wall thickness of 24 mm. No gadolinium-based contrast agent was given, owing to needle phobia. CMR imaging and echocardiography also showed a dilated and tortuous-looking left anterior descending (LAD) coronary artery. A computed tomographic (CT) angiogram 2 months prior to the event showed an unusually large left mainstem and proximal LAD but no anomalous connections or coronary artery aneurysms.0000000000000000

The boy was generally fit and well with no previous history of syncope, but he had occasional palpitations and mild chest pain when playing sports. There was no family history of cardiac disease. Because he was under pediatric cardiology follow-up at our institution, he was seen in our clinic 2 days prior to the event, where, for risk stratification, he underwent exercise testing and was fitted with the Holter monitor. This showed progression from sinus rhythm to VF (Fig. 1) at the time of the event.

Immediately following the collapse at 14:20, a teacher and two first aid workers carried out an initial assessment with the patient in the recovery position, during which he was breathing. Five minutes later, responsiveness and breathing deteriorated, and CPR was started. Paramedics arrived approximately 10 min from the moment of collapse and resumed CPR. The paramedics delivered two direct current defibrillator shocks for VF, following which the patient reverted to sinus rhythm with a total downtime of 24 min.

At the time of the event at 14:21, the boy’s heart rate rose to a maximum of 168 beats/min with an increasing number of ectopic beats and soon changed to torsades de pointes and degenerated into VF. Prior Holter recordings were unremarkable with no evidence of non-sustained VT or arrhythmia. The episode of VF lasted for 24 min before the boy was reverted back into sinus rhythm.

He was intubated and transferred to a district general hospital, where he scored 3 on the Glasgow Coma Scale with decorticate posturing. Cranial CT showed cerebral edema. Neuroprotective measures were put in place and upon discussion with our tertiary center. He was commenced on amiodarone and transferred to our specialist pediatric intensive care unit (PICU). Upon arrival, he was sedated, paralyzed, and ventilated on bilevel positive airway pressure. Antiarrhythmic treatment initially included amiodarone (infusion rate 5 μg/kg/min) and bisoprolol. Neuroprotective measures were continued for 72 hr due to bradycardia (40 beats/min) with hypertension indicating raised intracranial pressure. A course of intravenous co-amoxiclav 500/125 mg every 8 hr was started due to left lower lobe chest radiographic changes suspicious of aspiration pneumonia with a maximum C-reactive protein of 60 mg/dl. The patient’s pupils were equal and reactive with no clinical or electrical seizure activity noted. Six days after admission, he was extubated and started mobilizing around his bed within 1 day. He made a full recovery with only minimal neurologic sequelae.

### Investigations

Echocardiography performed upon admission showed preserved biventricular systolic function (ejection fraction 79%) with no obvious regional wall abnormalities and diastolic dysfunction. His troponin I level upon admission to the PICU was 550 ng/L (normal range, < 40 ng/L), which dropped to 53 ng/L after 48 hr (normal range, < 40 ng/L), and his brain natriuretic peptide (BNP) level was 325 ng/L (normal range, < 20 ng/L). Results of his renal and liver function tests were normal. At discharge from PICU, the patient was alert but not fully oriented to time and space, with short-term memory impairment but appropriate interaction. The result of his cranial nerve examination was normal. Tone in all four limbs was good, and his movement was symmetrical, but his power was slightly reduced. He had no clonus. His deep tendon reflexes were elicited, and he had ataxic gait with some slurring of speech. The findings of brain magnetic resonance imaging performed 7 months after the event were normal. Blood tests done 6 months later showed his BNP level was 154 ng/L, and his troponin I level was within the normal range.

### Treatment

Anti-arrhythmic treatment with bisoprolol (3.75 mg once daily) was continued. In addition, ICD implantation was performed for secondary prevention. Neurological follow-up examinations did not detect any neurological deficits. A care plan for school was developed by pediatric cardiomyopathy nurse specialists, including recommendations for limitation of sporting activities.

### Outcome and follow-up

This 9-year-old boy, although he had some mild deterioration in his reading, remains at the top of the class in his primary school following an OHCA due to VF. In the 6 months following this episode, there were no subsequent ICD discharges, and overall he was able to recover from the VF arrest without any lasting brain damage and was able to return to moderate exercise.

He will need regular lifelong follow-up, both with device specialists and with cardiologists with expertise in cardiomyopathy, currently scheduled every 3 months. He has been advised to receive endocarditis prophylaxis for dental or surgical procedures with regard to his ICD and complex cardiac disease.

## Discussion and conclusion

NSML, an autosomal dominant condition first described by Gorlin *et al*. in 1969 [4], presents with cardiac abnormalities and can cause sudden cardiac arrest, most likely secondary to extensive thickening of the ventricular wall and possibly fibrosis. Our patient presented with severe asymmetric LV hypertrophy with a maximum wall thickness of 24 mm on CMR imaging. This finding is in agreement with a study by Limongelli *et al.*, who examined a large series of patients (n = 26) with NSML and found that LV hypertrophy was present in 73% of all patients, with most of them having asymmetric LV hypertrophy. LV outflow tract obstruction and abnormal mitral filling patterns were found in half of the patients, and 30% had right ventricular hypertrophy [3]. Although it is evident that severe HCM confers a high risk for SCD even in children, the question whether concomitant syndromes confer an additional risk is yet to be fully investigated. Additionally, what remains to be answered is whether SCD is predictable in NSML and whether prophylactic ICD implantation should be considered not only in primary HCM but also in HCM secondary to NSML and other conditions such as Friedreich’s ataxia, Barth syndrome, and other mitochondrial and metabolic conditions. Due to the rarity of NSML, there is a lack of data with regard to the progression of the disease and risk of SCD. Further work needs to be performed to correlate the occurrence of adverse cardiovascular events with specific *PTPN11* gene mutations [5, 6] and non-*PTPN11* gene mutations in order to establish better risk stratification guidelines.

The National Australian Childhood Cardiomyopathy Study (NACCS) reported that in patients with HCM, the presence of Noonan syndrome conveyed an increased risk of poor outcome with a hazard ratio of death or transplant of 2.9 [7]. The North American Pediatric Cardiomyopathy Registry also reported that in patients with HCM, Noonan syndrome was associated with worse survival [8]. Bharucha *et al*. [9], focusing on sudden death in children with cardiomyopathy in NACCS, reported that 16,289 (5.5%) eligible patients experienced SCD over follow-up of nearly 12 years. The most important risk factor for SCD in the HCM subgroup (n = 80) was the extent of LV posterior wall thickness.

Our case report is partly in agreement with Limongelli *et al.*, who reported a case of a 9-year-old boy with NSML and SCD. Unlike our patient, their patient had a non-sustained seven-beat run of VT recorded on a Holter ECG and did not show progression to VF; even though ICD implantation was agreed, their patient died suddenly while walking home from shopping before ICD implantation could take place [2]. Woywodt *et al.* observed a 26-year-old man with NSML who was resuscitated from VT in the absence of severe LV hypertrophy and, similar to our case, prior arrhythmias [3]. Although Woywodt *et al*. [3] emphasized that their case report was the first report of survival from VF in a patient with NSML, their report explained little about post-cardiac arrest care or complications.

In summary, our case report highlights that patients with NSML and LV hypertrophy are at an increased risk of adverse events that may occur without prior warning or ventricular arrhythmias. A higher likelihood of survival in cardiac arrests of complex cardiac patients is achieved through effective bystander CPR, prompt involvement of emergency services, and referral to specialist centers, as demonstrated by our case report. Though evidence-based data are lacking, ICD implantation may be considered for primary prevention of SCD in these patients after a careful and individualized benefit-to-risk assessment.

## Abbreviations

BNP: Brain natriuretic peptide
CMR: Cardiac magnetic resonance
CPR: Cardiopulmonary resuscitation
CT: Computed tomography
ECG: Electrocardiogram
HCM: Hypertrophic cardiomyopathy
ICD: Implantable cardioverter defibrillator
LAD: Left anterior descending
LV: Left ventricular
NACCS: National Australian Childhood Cardiomyopathy Study
NSML: Noonan syndrome with multiple lentigines
OHCA: Out-of-hospital cardiac arrest
PICU: Pediatric intensive care unit
SCD: Sudden cardiac death
VF: Ventricular fibrillation
VT: Ventricular tachycardia
